# Immediate Implant Placement in the Maxillary Aesthetic Zone: A Cone Beam Computed Tomography Study

**DOI:** 10.3390/jcm10245853

**Published:** 2021-12-14

**Authors:** Anna Botermans, Anna Lidén, Vinícius de Carvalho Machado, Bruno Ramos Chrcanovic

**Affiliations:** 1 Faculty of Odontology, Malmö University, 214 21 Malmö, Sweden; anna-botermans@hotmail.com (A.B.); anna-liden@hotmail.com (A.L.); 2 Slice Diagnóstico Volumétrico por Imagem, Belo Horizonte 30140-110, Brazil; viniciusdecmachado@hotmail.com; 3 Department of Prosthodontics, Faculty of Odontology, Malmö University, 214 21 Malmö, Sweden

**Keywords:** dental implant, immediate implant placement, virtual treatment planning, anterior maxilla, cone beam computed tomography, fenestration, risk assessment

## Abstract

This study aimed to investigate the factors that could be associated with the risk of labial cortical bone wall perforation with immediate implant placement (IIP) in the maxillary aesthetic zone, in a cone-beam computed tomography (CBCT) virtual study. CBCT exams from 126 qualified subjects (756 teeth) were included. Implants were virtually positioned in two different positions: in the long axis of the tooth (prosthetically-driven position) and in an ideal position in relation to adjacent anatomical structures (bone-driven position). Two different implant diameters were planned for each tooth position, namely, 3.75 and 4.3 mm for central incisors and canines, and 3.0 and 3.3 mm for lateral incisors. The incidence of perforation was nearly 80% and 5% for prosthetically- and bone-driven position, respectively. Factors associated with a higher risk of cortical bone wall perforation (bone-driven position), according to logistic regression analysis, were women, wider implants, Sagittal Root Position class IV, and decrease of the labial concavity angle. Perforation of the labial cortical bone wall can be greatly minimized when the implant is placed in a bone-driven position compared to a prosthetically-driven position. It is important to preoperatively evaluate the morphological features of the implant site for risk assessment and to individualize the treatment plan.

## 1. Introduction

According to the first well-established protocol for the modern dental implants, implants were surgically placed in the jaws only after a 3–6-month period after tooth extraction, to ensure satisfactory remodeling and healing of the alveolar bone in order to optimize osseointegration of the implant [1]. Nowadays, it has been widely accepted that dental implants can be inserted into the alveolar socket immediately after extraction of teeth, with survival rates comparable to the ones observed for implants placed in pristine or healed sites [2,3]. The findings of a systematic review on the subject, gathering together data from 73 studies, showed a failure rate of 4.00% (330 failures out of 8241 implants) for implants placed in fresh extraction sockets in comparison to 3.09% (599 failures out of 19,410 implants) for implants placed in healed sites [2]. According to the results of another review [3], the survival rates are high even when implants are immediately placed in sockets with periodontal or endodontic infection, with studies observing a minimum survival rate of 85.7%, reaching 100% in many studies.

The immediate implant placement (IIP) approach has its particularities depending on which region of the jaws is involved. The alveolar bone of the anterior maxilla is usually proclined in an anterior-inferior direction, forming a concavity right above the apical region of the teeth, and the labial bone is this region is usually thin [4]. Therefore, it is expected that the frequency of bone dehiscence and esthetic problems would be higher in this region in comparison to the posterior regions of the jaw [5], as the alveolar ridge is followed by a great reduction of its dimensions after the extraction of a tooth [6]. The issue is especially important if the implants planned to be placed immediately after extraction are positioned in the same position as the tooth that previously occupied the socket [7], the so-called prosthetically-driven position, as it is advocated that the implant should be anchored by a minimum amount of bone apical to the socket in order to achieve primary stability [8,9]. Having that in mind, it is important that an adequate pre-treatment evaluation is conducted in the cases for which IIP is planned [10].

Previous CBCT studies assessing IIP in the anterior maxilla focused on the possibility of having straight-channel screw-retained single crowns [11], on the likelihood of needing facial bone augmentation due to the morphology of the alveolar ridge [7], on evaluating alveolar ridge dimension and the presence labial undercut [12], on determining the thickness of the facial bone wall and the sagittal angulation between the axis of the teeth and the long axis of the associated alveolar bone [13], on assessing the labial bone perforation when implants were planned along the palatal slope of the investigated tooth root [14], and on investigating the risk of bone fenestration based on labial bone thickness [15]. All these studies call attention to the fact that there is a considerable risk of labial bone perforation when IIP are planned for the maxillary esthetic zone.

However, as far as the authors of the present study are aware, there is no study comparing the prevalence of labial cortical bone perforation between the bone- and prosthetically-driven ideal position in the anterior maxilla for IIP, or even only the incidence of perforation for implants immediately inserted in extraction sockets at the anterior maxilla when these are placed in a bone-driven ideal position. Therefore, the purposes of the present cone beam computed tomography (CBCT)-scan virtual planning study was (1) to determine the risk of labial bone plate perforation when implants are virtually planned along the longitudinal axis of the tooth in three maxillary teeth areas (central incisor—CI, lateral incisor—LI, and canine—CA) and in a bone-driven position, in case of immediate implant placement; (2) to determine the minimal implant length possible without perforation, while respecting a secure distance from adjacent anatomical structures; (3) to determine the angle between the implants in the two aforementioned positions; and (4) to assess possible associations between all the covariates and perforation of the labial bone plate when the implant is planned in the ideal bone-driven position. The null hypothesis of the present study was that there would not be a significant difference in the prevalence of cortical bone perforation between bone-driven and prosthetically-driven ideal position for IIP placed in the anterior maxilla, against the alternative hypothesis of a difference.

## 2. Materials and Methods

### 2.1. Subjects

The present retrospective analysis was based on the maxillary scans performed in Slice Diagnóstico Volumétrico por Imagem, in Belo Horizonte, Brazil, during the last quarter of the year 2014. The scans used in the present study were selected from the CBCT database and were not specifically acquired for this publication.

### 2.2. Ethical Considerations

The study was approved by the local Ethics Committee (PUC-MG, Belo Horizonte, Brazil). The patients were contacted through a telephone call, and a signed informed and written consent form was obtained from each patient approving the use of their scans. The patients were not identifiable in any way, and a decoding list linking patient names and numbers was used and stored by the principal investigator, which was destroyed after completion of the study. The investigation was conducted according to the principles embodied in the Helsinki Declaration of 1964 for biomedical research involving human subjects, as amended in 2013.

### 2.3. Inclusion and Exclusion Criteria

The following inclusion criteria were applied: (a) CBCT examinations from patients who allowed use of their scans; (b) CBCT examinations of the maxilla; (c) presence of fully erupted bilateral maxillary CI, LI, and CA; (d) each tooth had to have fully formed apexes; and (e) each tooth had to be normally positioned and have normal alignment, with harmonious incisal line across the maxillary anterior teeth.

CBCT examinations were excluded on the basis of (a) the presence of technical artifacts that hindered the evaluation of the focused structures; (b) images that had an implant, a pathologic lesion, evident root resorption, or a missing tooth; and (c) examinations from patients that had a history of orthodontic treatment, orthognathic surgical surgery, grafted alveolar ridge, supernumerary or impacted teeth, preexisting alveolar bone destruction, perforation, dehiscence, or a combination of these caused by periodontal disease or traumatic injury around the investigated region.

### 2.4. Hardware and Software

CBCT scanning was performed with an i-CAT CBCT system (Imaging Sciences International, Hatfield, PA, USA). The scans were acquired using the i-CAT 3D Imaging System (i-CAT Vision Software, Imaging Sciences International, Hatfield, PA, USA) and included the entire maxilla. The following CBCT scan parameters were used for all patients: a tube voltage of 110 kV, 1 to 20 mA, emission of x-rays over an interval of 40 s, and an effective dose of 136 μSV. Measurements were obtained on the transversal sections of the selected teeth, with the use of a computer software (DentalSlice Navegação Virtual, Bioparts, version 2017, Brasília, Brazil). The distance between the obtained transversal sections were 1.0 mm, and the voxel size 0.2 mm. The field of view (FOV) was standard (medium; 6 × 14 cm), capturing the entire maxilla.

### 2.5. Sample Size Calculation

The calculation of the sample size was based on the results of the study of Zhou et al. [7], which observed an incidence of 26.1% of fenestration (labial cortical bone perforation) for IIP planned for the anterior maxilla, in a prosthetically-driven ideal position. Although one study assessed the labial bone perforation when implants were planned along the palatal slope of the investigated tooth root [14], this is not the same as the bone-driven ideal position considered for the present study. As there is no previous study comparing the prevalence of labial cortical bone perforation between the bone- and prosthetically-driven ideal position in the anterior maxilla for IIP, or even only the incidence of perforation for implants immediately inserted in extraction sockets at the anterior maxilla when these are placed in a bone-driven ideal position, it was hypothesized that this figure would be four times as much in comparison to IIP placed in a bone-driven ideal position. Therefore, having an anticipated fenestration incidence of 26% and 6.5% for prosthetically- and bone-driven ideal position, respectively, there was a need of 55 cases in each group having set alpha (α) at 0.05 and power at 80%. The sample size calculation was performed with ClinCalc.com.

### 2.6. Definitions and Measurements

Calibration between three authors (A.B., A.L., and B.R.C.) was performed prior to the study in 10 CBCT exams, concerning the position of the implants and all the measurements. After that was done, the measurements were conducted by the two first authors (A.B., A.L.) of the manuscript.

#### 2.6.1. Sagittal Root Position (SRP)

The teeth were classified into SRP classes with respect to the anterior maxillary osseous housing, according to a previous study [16]:

- Class I: the root is positioned against the labial cortical plate;
- Class II: the root is centered in the middle of the alveolar housing without engaging either the labial or the palatal cortical plates at the apical third of the root;
- Class III: the root is positioned against the palatal cortical plate;
- Class IV: at least two-thirds of the root is engaging both the labial and palatal cortical plates.

#### 2.6.2. Secure Distance from Implant to the Adjacent Anatomical Structures

Implants were placed according to a secure distance from adjacent anatomical structures. In the maxillary aesthetic zone, these were adjacent teeth, floor of nasal sinus, floor of maxillary sinus, nasopalatine nerve canal, and labial and palatal cortical bone plates. Distance between the implant and the structures for CI, LI, and CA is defined as the distance between the closest point of the implant to the aforementioned structures. The minimum distance between the implant and the adjacent tooth was established as 2 mm, according to the recommendations that this distance should not be shorter than 1.5 to 2 mm [17]. The minimum distance between the implant apex to the nasal floor was established at 2 mm. Moreover, a 2-mm secure distance was kept from all external cortical bone plates.

#### 2.6.3. Implant Simulation

Bucco-lingually, the center of the implant platform was positioned along an imaginary line along the long axis of the tooth. A parallel implant was selected for virtual IIP. The subjects were divided into two equally large groups and virtually received implants with different diameters. One half received larger diameters, of 4.3 mm, 3.3 mm, and 4.3 mm for CI, LI, and CA, respectively. The other half received narrower implants with the diameters of 3.75 mm, 3.0 mm, and 3.75 mm for CI, LI, and CA, respectively. The cases were randomized by using the RAND function in Excel software (Microsoft Co., Redmond, WA, USA).

For all the simulated implants, the implant platform was positioned 1 mm below the buccal crestal level, in order to follow the approximated 3-year mean marginal bone loss for implants placed immediately in the anterior maxilla [18]. Moreover, the minimal amount of bone apical to the alveolar socket apex requiring one to achieve primary stability has been considered to be 4 mm to minimize the risk of early implant loss [8,9].

**Figure 1:**
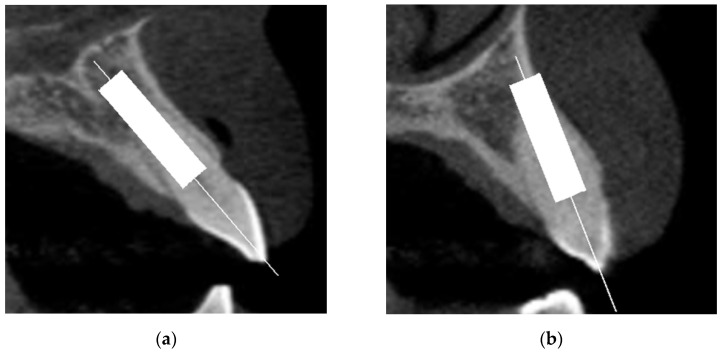
Absence (**a**) and occurrence (**b**) of labial bone plate perforation.

In each tooth site, implants were positioned in two ways:

(a) Prosthetically-driven ideal position: implant placed along the long axis of the tooth root and crown along line A shown in the sagittal section (line A was defined as the line connecting the incisal edge and the root apex of the tooth, bisecting the labial and palatal halves of the tooth), with the implant anchored in at least 4 mm of native bone. Depending on the case, this could lead to absence (Figure 1a) or occurrence (Figure 1b) of labial bone plate perforation. In absence of perforation, it was also noted if the implant respected the 2 mm distance to adjacent anatomical structures. The proper mesio-distal angulation was verified in the panoramic view;
(b) Bone-driven ideal position: the minimal implant length possible without perforation, when anchoring the implant apex with 4 mm of native bone, still respecting the minimum 2 mm distance from the nasal floor and from the labial and palatal bone plates. The proper mesio-distal angulation was verified in the panoramic view.

#### 2.6.4. Implant-Line A Angle (ILAA)

**Figure 2:**
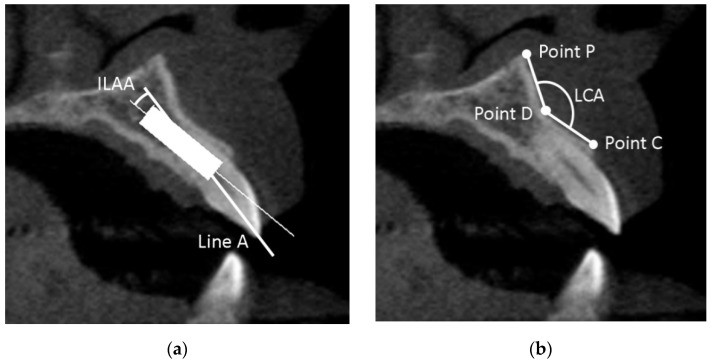
(**a**) The implant-line A angle (ILAA); (**b**) the labial concavity angle (LCA).

The angle between the prosthetically-driven ideal position (line A) and the long axis line of the implant in bone-driven position was determined, and defined as the implant-line A angle (ILAA) (Figure 2a).

#### 2.6.5. Labial Concavity Angle (LCA)

The LCA was defined as the angle between the line D–C and the line D–P (Figure 2b). Point C was defined as the most coronally external point of the labial plate, point D as the most internal point, and point P as the most external apical point of the labial plate superior to point D.

#### 2.6.6. Angle Measurement

The images generated were later transferred to the Image J software version 1.8.0_172 (National Institute of Health, Bethesda, MD, USA) in order to measure the angles involved in the study.

### 2.7. Statistical Analyses

The data were statistically analyzed using the SPSS version 27 software (SPSS Inc., Chicago, IL, USA). The mean, standard deviation, minimum, and maximum for each of the measurements were calculated. Variations were evaluated according to the tooth (CI, LI, CA), the predictor variable. The other variables were the maxillary side (left/right), age, and sex. Kolmogorov–Smirnov test was performed to evaluate the normal distribution. Levene’s test evaluated homoscedasticity. Paired *t*-test and Wilcoxon test, where indicated, were performed to compare the measurements of each tooth between the left and right side of the maxilla. The performed tests for the comparison of independent groups (tooth, sex) were Student’s *t*-test or Mann–Whitney test, depending on the normality. Comparison of three or more groups was performed with one-way ANOVA or Kruskal–Wallis test, depending on the distribution. Pearson’s chi-squared test or Fisher’s exact test were used for categorical variables, depending on the expected count of events in a 2 × 2 contingency table. Pearson correlation and linear regression were performed to verify the relationship between the patients’ age and the LCA, the ILAA, and the minimal implant length possible. Spearman correlation was performed to check the relationship between the sex and the SRP, the LCA, the ILAA, and the minimal implant length possible.

Univariate and multivariate binary logistic regression was used to assess possible associations between all the covariates and perforation of the labial bone plate when the implants were planned in the prosthetically-driven ideal position. Odds ratio (OD) and 95% confidence intervals (95% CI) were estimated from the regression models.

For the final multivariate regression model, only the variables that were moderately associated (*p* < 0.10) with perforation of the labial bone plate and did not present multicollinearity were included. In order to verify multicollinearity, a correlation matrix of all of the predictor variables with a significant OD (*p* value cut-off point of 0.1) identified in the univariate models was scanned, to see whether there were some high correlations among the predictors. Collinearity statistics obtaining variance inflation factor (VIF) and tolerance statistic were also performed to detect more subtle forms of multicollinearity.

The degree of statistical significance was considered *p* < 0.05.

## 3. Results

### 3.1. Selection of Cases

From the 574 CBCT exams of the maxilla performed at the aforementioned oral radiology company during the last quarter of 2014, 414 exams were initially excluded either due to one or more missing teeth in the focused area, from patients that had a history of or were under orthodontic treatment, or due to the presence of anterior maxillary teeth with misalignment. Of the remaining 160 CBCT exams, 34 were excluded either due to the presence of radiological artefacts that hindered the evaluation of the focused structures, bone destruction in the apical region of one or more teeth, less often due to low marginal bone level, or a combination of these. The remaining 126 CBCT exams were equally and randomly allocated between the two groups of different implant diameter, namely, 3.0/3.75 mm (narrower implants) and 3.3/4.3 mm (wider implants).

### 3.2. Description of the Cohort Group

The description of the cohort group is shown in Table 1. The groups did not statistically significant differ concerning the distribution of individuals of different sexes, the mean age between males and females in the same group of implant diameter, or between males or females in different groups of implant diameter.

### 3.3. Measurements

Table 2 shows the distribution of teeth according to the SRP classes. It can be noticed that most of the teeth presented the root positioned against the labial cortical plate (class I), followed by teeth with most part of their roots engaging both the labial and palatal cortical plates (class IV), the latter was more common for lateral incisors. The root of the teeth was rarely positioned against the palatal cortical plate (class III), occurring only in lateral incisors.

When planning the placement of implants in bone-driven position in the CI tooth region, the nasopalatine canal was perforated in 43.7% (55/126) of the cases in the narrower implants group and in 61.1% (77/126) of the cases in the wider implants group (*p* = 0.006, Pearson’s chi-squared test). Regardless of diameter, 52.4% (132/252) of the implants in tooth region CI perforated the nasopalatine canal.

The mean minimum length of the planned implants when in bone-driven position, without perforation or invasion of the 2 mm secure distance from the surrounding anatomical structures, is shown in Table 3. The mean values were statistically significant different for implants planned in tooth region 21. The mean value increased from CI to LI and then to CA. The difference of the mean values was statistically significant different between the three groups of teeth (*p* < 0.001, Kruskal–Wallis test; *p* < 0.001, Kolmogorov–Smirnov test). The Dunn’s post hoc test with Bonferroni correction showed that the difference was statistically significant between all direct comparisons, namely, between CI and LI (*p* = 0.012; Levene’s test: *p* = 0.966), between CI and CA (*p* < 0.001; Levene’s test: *p* = 0.810), and between LI and CA (*p* < 0.001; Levene’s test: *p* = 0.849).

Table 4 shows the values for the LCA according to the different tooth positions, as well as for the different sexes. There was no statistically significant difference of LCA mean value between the groups (*p* = 0.374, one-way ANOVA; *p* = 0.078, Kolmogorov–Smirnov test), when all measurements where considered. There was a very weak correlation between LCA and sex of the individuals (*r*_s_ = −0.056, *p* = 0.125; Spearman correlation), as well as between LCA and age (*r* = 0.065, *p* = 0.075; Pearson correlation) and between LCA and SRP class (*r*_s_ = −0.189, *p* < 0.001; Spearman correlation).

Table 5 shows the frequency of cortical bone perforation for the narrower 3.0/3.75 mm implants, for both prosthetically- and bone-driven positions, and the ILAA. It can be observed that the frequency of perforation is higher when the implants are planned in the prosthetically-driven position in relation to implants planned in the bone-driven positions. The difference of the prevalence of cortical bone perforation between prosthetically- and bone-driven ideal position, irrespective of implant diameter, was highly statistically significant (*p* < 0.001, Pearson’s chi-squared test). There were only three cases (out of 378) without perforation, for the prosthetically-driven implants. The mean ILAA angle was determined 17.7 ± 7.2 degrees, irrespective of implant diameter. The mean ILAA increased from central incisors, to canines, and to lateral incisors, which showed the higher mean values. There was a statistically significant difference for the mean ILAA values when the three groups of teeth (CI, LI, CA) were compared (*p* = 0.036, Kruskal–Wallis test; *p* = 0.046, Kolmogorov–Smirnov test). The Dunn’s post hoc test with Bonferroni correction showed that the statistically significant difference for the mean ILAA values lay in the comparison between CI and LI (*p* = 0.033; Levene’s test: *p* = 0.070), with no significance between the other direct comparisons, namely, between CI and CA (*p* = 1.000; Levene’s test: *p* = 0.795) and between LI and CA (*p* = 0.245; Levene’s test: *p* = 0.176).

Table 6 shows the frequency of cortical bone perforation for the wider 3.3/4.3 mm implants, for both prosthetically- and bone-driven positions, and the ILAA. As observed for the narrower implants, the frequency of perforation is higher when the implants are planned in the prosthetically-driven position in relation to implants planned in the bone-driven positions. There was only one case (out of 378) without perforation, for the prosthetically-driven implants. The mean ILAA increased from central incisors, to canines, and to lateral incisors, which showed the higher mean values. There was a statistically significant difference for the mean ILAA values when the three groups of teeth (CI, LI, and CA) were compared (*p* = 0.006, Kruskal–Wallis test; *p* = 0.002, Kolmogorov–Smirnov test). The Dunn’s post hoc test with Bonferroni correction showed that the statistically significant difference for the mean ILAA values lay in the comparison between CI and LI (*p* = 0.013; Levene’s test: *p* = 0.206), as well as in the comparison between CI and CA (*p* = 0.022; Levene’s test: *p* = 0.992), but not for the comparison between LI and CA (*p* = 1.000; Levene’s test: *p* = 0.213).

When the groups of implants of different diameter were compared, it was possible to virtually place more 3.0/3.75 mm implants without perforation of the cortical bone and respecting the minimum secure distance of 2 mm from surrounding anatomical structures (217/378) than 3.3/4.3 mm implants (158/378), in the bone-driven position (*p* < 0.001; Pearson’s chi-squared test).

The correlation between the angles ILAA and LCA was very weak (*r* = −0.004, *p* = 0.945; Pearson correlation), as well as between ILAA and SRP class (*r*_s_ = −0.167, *p* = 0.001; Spearman correlation), and between ILAA and sex (*r*_s_ = −0.095, *p* = 0.065; Spearman correlation). The correlation between ILAA and age was weak (*r* = 0.256, *p* < 0.001; Pearson correlation).

Patients’ sex, tooth region, implant diameter, SRP class, and LCA were the factors identified by the univariate binary logistic regression models to possibly have an influence on the occurrence of the cortical bone perforation or invasion of the 2 mm secure distance from the surrounding anatomical structures by the planned implant in bone-driven position (Table 7), with patients’ sex (female), implant diameter (wider implants), SRP class (in relation to class 1) and LCA (decrease of the angle) remaining statistically significant in the multivariate model (Table 8).

## 4. Discussion

The results of the present study showed that the difference of the prevalence of cortical bone perforation between prosthetically- and bone-driven ideal position, irrespective of implant diameter, was highly statistically significant. The null hypothesis was therefore rejected.

SRP class I, when the root is positioned against the labial cortical plate, was by far the most prevalent SRP class observed in the study. This suggests that implants that are planned to be immediately placed in the anterior maxilla will, in most cases, need to have their coronal part tilted labially in order to get enough anchorage of the available bone apical to the alveolar socket. As a clinical implication of this result, most of the implant-supported single crowns would need to be cemented on a custom-made prosthetic abutment, with the latter having a mean labial-palatal angulation of nearly 18 degrees, reflecting the mean ILAA, which was of 17.7 ± 7.2 degrees. Another alternative prosthetic solution would be the use of individualized abutments with an angled screw channel [19], making it possible to restore the implant with a screw-retained crown instead. Considerable attention should be given to this, as the results of a recent study showed that it was possible to use straight-channel screw-retained single crowns in only 14% of the implants planned to be immediately placed in the maxillary esthetic zone [11]. The second most prevalent SRP class observed in the study was class IV, in which at least two-thirds of the root engaged both the labial and palatal cortical plates [16]. This meant that there was virtually no bone left in the alveolar socket after the tooth was extracted, only apical to it. Among the anterior teeth, class IV was more often observed for lateral incisors. This is related to the restricted bone volume usually found in this region [20]. This means that even greater attention is needed when IIP is planned for maxillary lateral incisors. Sung et al. [14] observed SRP class I as the class with the higher occurrence of perforation despite having observed similar rates of SRP class I to the current study. Their [14] frequency distribution of SRP class II and class IV differed from the current and another study [16], while our results were more similar to latter [16].

Perforation of the nasopalatine canal was observed in about half of the implants planned for the CI position. Even though it was not possible to place 4.3 mm implants in a bone-driven position in the central incisor sockets respecting the 2 mm safety margin from the surrounding anatomical structures in more than half (61.1%) of the cases, the choice for implants of narrower diameter in these cases would still encounter the nasopalatine canal in a considerable number of cases (43.7%). However, this is not considered an impairment for this kind of procedure, as shown by some studies [21,22]. It is recommended that the contents of the canal be curetted out before the placement of an implant [22], which usually results in sensory disorders in the anterior palatal region. However, this sensation usually recovers after a couple of months through the compensatory action of the branches of the greater palatine nerves [23]. Bleeding of nasopalatine artery, however, is something that the operator needs to be aware of [24], as well as a possible additional difficulty when placing the implant in the planned position. It is worth mentioning that the nasopalatine canal is not the only anatomical structure carrying neurovascular structures in the area. The presence of accessory canals of the canalis sinuosus is also something important to take into consideration when implants are planned to be placed in the anterior maxilla [25].

Primary stability is of the utmost importance in order to obtain a successful outcome regardless of the timing of implant placement in relation to tooth extraction. One of the assumed crucial factors in order to obtain primary stability when implementing IIP is a minimum of 4 mm of apical anchorage [8,9]. With this in mind, a longer implant can be necessary when performing IIP versus conventional implant placement. According to this study, the minimum value of implant length in bone-driven position ranged from 7.25 mm to 17.5 mm in the maxillary aesthetic zone. The approximately 10 mm difference in length implied that there could be a large variation between subjects, meaning the required implant length was highly individual. It is therefore necessary to examine every individual separately. The length increased from central incisor to lateral incisor and then to canine. This means that in the clinical scenario there is not only a need to adapt the length between individuals, there is also a need to adapt the length according to tooth region.

The multivariate regression model identified four factors that increased the risk of the occurrence of cortical bone perforation or invasion of the 2 mm secure distance from the surrounding anatomical structures by the implant virtually placed in the bone-driven position. These factors were the patients’ sex, the implant diameter, the SRP class, and the LCA.

Women were 4.5 times more likely to present a bone perforation of the labial cortical bone plate than men. The discrepancy can possibly be linked to a general volume variation of the facial bones between the genders [26,27,28], which is something to hold in regard when implementing IIP.

The wider implant diameter posed an approximately doubled risk of perforation when placing the implant in the bone-driven position, compared to the narrow implants. A narrow implant can be considered a safer option as the risk of perforation is decreased and the 2 mm distance is respected. However, a narrower implant can forsake a larger gap between the alveolar socket walls and the implant. If fibrous tissue forms at the interface between bone and implant, the clinical outcome can possibly be compromised [29]. This emphasizes the importance of choosing an implant with suitable dimension and shape.

SRP class IV presented a significantly higher risk of perforation compared to the other classes. When the root engages both the labial and the palatal cortical bone plates, the amount of surrounding bone is limited. This limitation may hinder repositioning of the implant, increasing the occurrence of perforation. The alveolar ridge morphology should therefore be taken into account.

The probability of perforation was reduced by 2.3% for every 1-degree increase in the LCA. A smaller LCA will generate a deeper labial concavity. With less bone volume available in the labial-palatal dimension at the tip of the angle, the possibility to reposition the implant without perforation becomes more limited. The mean LCA was approximately 152 degrees, although a relatively large discrepancy of 73 degrees between maximum and minimum value was noted between the subjects.

As for limitations of the present study, it is important to stress that the validity of these results relies on the accuracy of CBCT images. Moreover, the measurements were based on single implant placement only, meaning the required distance between implants when more than one implant was placed adjacently was not taken into consideration.

Considering the large risk of labial bone perforation while implementing IIP in the maxillary aesthetic zone, meticulous pre-operative planning is crucial. A careful examination beforehand of the individual patient and the possible risk factors associated with IIP can provide essential data for the treatment planning in the anterior maxilla. Although 2-D radiographic exams are usually more accessible, present lower cost, and emit low radiation doses, they present limited information concerning pre-assessment of the risk of labial cortical bone perforation. 3-D imaging, such as CBCT, provides a sagittal-sectioned view that can provide essential added information to ensure a more optimal implant placement in the anterior maxillary osseous housing [14,30].

It is expected that the results of this study can be applied practically to any population, as CBCT exams of individuals from both sexes and with a wide age range were included, which not only strengthens the study, it also provides a good generalization. Further clinical studies with a larger sample size should be made to confirm the outcomes of the present investigation.

## 5. Conclusions

As conclusions of the present study, we can list:

- The mean minimum length of the planned implants when in bone-driven position, without perforation or invasion of the 2 mm secure distance from the surrounding anatomical structures, increased from CI to LI and then to CA;
- The incidence of perforation was nearly 80% and 5% for prosthetically- and bone-driven position, respectively;
- The mean angle between the tooth position and the corrected angulation in order to be able to install an implant in a safe manner with enough bone anchorage (angulation between the prosthetically-driven and the bone-driven position) was 17.7 ± 7.2 degrees;
- Factors associated with a higher risk of cortical bone perforation (in bone-driven position), according to logistic regression analysis, were women, wider implants, SRP class IV, and decrease of the labial concavity angle.

## Figures and Tables

**Table 1:** Description of the cohort group, according to the groups of implant diameter and sex.

	Narrower Implants (3.0/3.75 mm)	Wider Implants (3.3/4.3 mm)	*p* Value
Individuals (*n*)	63	63	
	Individuals/teeth (n)		
Male	27/162	24/144	0.586 ^a^
Female	36/216	39/234	0.182 ^b^
	Age, mean ± SD (min-max) (years)		
Male	50.4 ± 16.7 (15.7–83.0)	52.0 ± 14.4 (14.2–74.6)	0.651 ^c^
Female	51.3 ± 15.3 (21.2–78.4)	51.0 ± 13.5 (20.5–76.9)	0.767 ^c^
*p* value	0.760 ^d^	0.630 ^d^	

^a^ Comparison of the number of individuals of different sexes between implant groups, Pearson’s chi-squared test. ^b^ Comparison of the number of teeth from individuals of different sexes between implant groups, Pearson’s chi-squared test. ^c^ Comparison of the mean age of the individuals between groups of implant diameter, Mann–Whitney test. ^d^ Comparison of the mean age between groups of individuals of different sex, within the same group of implant diameter, Mann–Whitney test.

**Table 2:** Distribution of teeth according to SRP classes.

SRP Class	I	II	III	IV	Total
Tooth	*n* (%)				
13	110 (87.3)	2 (1.6)	0 (0)	14 (11.1)	126 (100)
12	87 (69.1)	11 (8.7)	3 (2.4)	25 (19.8)	126 (100)
11	109 (86.5)	9 (7.2)	0 (0)	8 (6.3)	126 (100)
21	112 (88.9)	8 (6.3)	0 (0)	6 (4.8)	126 (100)
22	93 (73.8)	9 (7.1)	2 (1.6)	22 (17.5)	126 (100)
23	113 (89.7)	1 (0.8)	0 (0)	12 (9.5)	126 (100)
Total	624 (82.5)	40 (5.3)	5 (0.7)	97 (11.5)	756 (100)

SRP—sagittal root position.

**Table 3:** Minimum length of the implants when planned in bone-driven position.

Tooth	3.0/3.75 mm	3.3/4.3 mm	*p* Value *
	mean ± SD (min, max)		
13	13.5 ± 1.8 (9.75, 17.5)	13.2 ± 1.3 (11.0, 16.5)	0.239
12	12.1 ± 1.6 (8.5, 15.0)	11.8 ± 1.5 (8.5, 15.5)	0.273
11	11.3 ± 1.8 (7.25, 15.0)	11.5 ± 1.9 (8.0, 16.0)	0.691
21	11.9 ± 1.8 (8.5, 15.0)	11.2 ± 1.3 (9.0, 14.9)	0.042
22	12.4 ± 2.0 (8.8, 16.0)	12.2 ± 1.7 (9.0, 15.5)	0.666
23	13.5 ± 1.7 (10.5, 16.5)	13.4 ± 1.7 (10.0, 16.0)	0.624
Global	12.4 ± 2.0 (7.25, 17.5)	12.3 ± 1.8 (8.0, 16.5)	0.229

* Comparison of the mean values between the groups of narrower and wider implants; Mann–Whitney test.

**Table 4:** LCA values—global and for the different sexes.

Tooth	LCA—Mean ± SD (Min, Max)			*p* Value *
	Global (*n* = 126 each tooth)	Male (*n* = 51 each tooth)	Female (*n* = 75 each tooth)	
13	152.0 ± 10.0 (105.3, 172.4)	152.4 ± 9.6 (105.3, 172.4)	155.8 ± 10.4 (126.1, 169.1)	0.960
12	151.3 ± 10.3 (123.7, 172.0)	151.8 ± 9.0 (132.6, 168.9)	151.0 ± 11.1 (123.7, 172.0)	0.911
11	152.8 ± 11.3 (117.2, 178.4)	153.1 ± 11.1 (129.5, 178.4)	152.7 ± 11.5 (117.2, 171.8)	0.927
21	152.8 ± 12.5 (117.5, 178.0)	154.0 ± 11.8 (132.0, 176.6)	152.0 ± 13.0 (117.5, 178.0)	0.581
22	151.0 ±10.5 (126.2, 177.5)	152.6 ± 8.3 (133.2, 170.1)	149.8 ± 11.7 (126.2, 177.5)	0.069
23	153.5 ± 8.6 (127.7, 174.4)	154.9 ± 6.8 (137.9, 166.2)	152.6 ± 9.6 (127.7, 174.4)	0.110
All teeth	152.2 ± 10.6 (105.3, 178.4) (*n* = 756)	153.1 ± 9.6 (105.3, 178.4) (*n* = 306)	151.6 ± 11.3 (117.2, 178.0) (*n* = 450)	0.125

LCA—labial concavity angle. SD—standard deviation. * Comparison of the LCA mean values between male and female individuals; Mann–Whitney test.

**Table 5:** Frequency of cortical bone perforation for 3.0/3.75 mm implants, for both prosthetically- and bone-driven positions, and the ILAA.

Tooth	Prosthetically Driven			ILAA	Bone Driven		
	No perforation	<2 mm	Perforation		No perforation	<2 mm	Perforation
		*n* (%)		mean ± SD (min, max)		*n* (%)	
13	0 (0)	12 (19.0)	51 (81.0)	17.3 ± 6.0 (7.0, 29.2)	35 (55.6)	20 (31.7)	8 (12.7)
12	0 (0)	17 (27.0)	46 (73.0)	19.0 ± 7.0 (5.0, 31.3)	33 (52.4)	29 (46.0)	1 (1.6)
11	1 (1.6)	13 (20.6)	49 (77.8)	16.5 ± 6.3 (0, 28.2)	39 (61.9)	23 (36.5)	1 (1.6)
21	0 (0)	14 (22.2)	49 (77.8)	16.0 ± 5.2 (2.2, 26.8)	41 (65.1)	21 (33.3)	1 (1.6)
22	1 (1.6)	15 (23.8)	47 (74.6)	19.4 ± 6.9 (0, 31.2)	32 (50.8)	30 (47.6)	1 (1.6)
23	1 (1.6)	7 (11.1)	55 (87.3)	17.2 ± 6.7 (0, 32.0)	37 (58.7)	17 (27.0)	9 (14.3)
Total	3 (0.8)	78 (20.6)	297 (78.6)	17.5 ± 6.4 (0, 32.0)	217 (57.4)	140 (37.0)	21 (5.6)

ILAA—Implant-line A angle.

**Table 6:** Frequency of cortical bone perforation for 3.3/4.3 mm implants, for both prosthetically- and bone-driven positions, and the ILAA.

Tooth	Prosthetically Driven			ILAA	Bone Driven		
	No perforation	<2 mm	Perforation		No perforation	<2 mm	Perforation
		*n* (%)		mean ± SD (min, max)		*n* (%)	
13	0 (0)	6 (9.5)	57 (90.5)	19.4 ± 7.2 (8.5, 35.5)	29 (46.0)	29 (46.0)	5 (8.0)
12	0 (0)	20 (31.7)	43 (68.3)	19.8 ± 10.3 (7.7, 45.4)	25 (39.7)	35 (55.5)	3 (4.8)
11	1 (1.6)	18 (28.6)	44 (69.8)	14.7 ± 7.7 (0, 29.5)	24 (38.1)	36 (57.1)	3 (4.8)
21	0 (0)	11 (17.5)	52 (82.5)	15.5 ± 7.2 (5.6, 33.3)	29 (46.0)	33 (52.4)	1 (1.6)
22	0 (0)	16 (25.4)	47 (74.6)	20.5 ± 7.6 (9.1, 38.9)	22 (34.9)	38 (60.3)	3 (4.8)
23	0 (0)	12 (19.0)	51 (81.0)	18.8 ± 8.1 (7.8, 38.9)	29 (46.0)	29 (46.0)	5 (8.0)
Total	1 (0.3)	83 (21.9)	294 (77.8)	18.1 ± 8.2 (0, 45.4)	158 (41.8)	200 (52.9)	20 (5.3)

ILAA—Implant-line A angle.

**Table 7:** Univariate binary logistic regression models for cortical bone perforation or invasion of the 2 mm secure distance from the surrounding anatomical structures (in relation to no perforation), for bone-driven implant position.

Factor	Odds Ratio (95% CI)	*p* Value
Sex		
Male	1	
Female	3.971 (2.913, 5.413)	<0.001
Age	1	
Increase by 1 year	0.995 (0.986, 1.005)	0.341
Tooth region		
Central incisor	1	
Lateral incisor	1.397 (0.984, 1.984)	0.062
Canine	1.040 (0.733, 1.476)	0.825
Implant diameter		
3.0/3.75 mm	1	
3.3/4.3 mm	1.877 (1.406, 2.505)	<0.001
SRP class		
1	1	
2	0.999 (0.525, 1.899)	0.997
3	1.831 (0.304, 11.034)	0.509
4	12.054 (5.728, 25.368)	<0.001
ILAA	1	
Increase by 1 degree	0.756 (0.531, 1.076)	0.120
LCA	1	
Increase by 1 degree	0.968 (0.955, 0.982)	<0.001

95% CI—95% confidence interval; ILAA—implant-line A angle; LCA—labial concavity angle; and SRP class—sagittal root position class.

**Table 8:** Multivariate binary logistic regression model for cortical bone perforation or invasion of the 2 mm secure distance from the surrounding anatomical structures (in relation to no perforation), for bone-driven implant position.

Factor	Odds Ratio (95% CI)	*p* Value
Sex		
Male	1	
Female	4.547 (3.229, 6.402)	<0.001
Tooth region		
Central incisor	1	
Lateral incisor	1.148 (0.767, 1.718)	0.502
Canine	0.966 (0.651, 1.433)	0.864
Implant diameter		
3.0/3.75 mm	1	
3.3/4.3 mm	2.064 (1.489, 2.860)	<0.001
SRP class		
1	1	
2	0.991 (0.491, 2.001)	0.979
3	0.536 (0.085, 3.391)	0.507
4	14.558 (6.601, 32.108)	<0.001
LCA	1	
Increase by 1 degree	0.977 (0.962, 0.993)	0.004

95% CI—95% confidence interval; LCA—labial concavity angle; and SRP class—sagittal root position class.

## Data Availability

The data presented in this study are available within the article.
